# High-efficiency quantitative control of mitochondrial transfer based on droplet microfluidics and its application on muscle regeneration

**DOI:** 10.1126/sciadv.abp9245

**Published:** 2022-08-17

**Authors:** Jiayu Sun, Hiu Tung Jessica Lo, Lei Fan, Tsz Lam Yiu, Adnan Shakoor, Gang Li, Wayne Y. W. Lee, Dong Sun

**Affiliations:** ^1^ Department of Biomedical Engineering, City University of Hong Kong, Hong Kong, Hong Kong SAR, China.; ^2^ Department of Orthopaedics and Traumatology, Faculty of Medicine, The Chinese University of Hong Kong, Prince of Wales Hospital, Shatin, Hong Kong SAR, China.; ^3^ Li Ka Shing Institute of Health Sciences, The Chinese University of Hong Kong, Shatin, Hong Kong SAR, China.; ^4^ Stem Cells and Regenerative Medicine Laboratory, Lui Che Woo Institute of Innovative Medicine, The Chinese University of Hong Kong, Prince of Wales Hospital, Shatin, Hong Kong SAR, China.; ^5^ SH Ho Scoliosis Research Laboratory, Joint Scoliosis Research Centre of the Chinese University of Hong Kong and Nanjing University, Department of Orthopaedics and Traumatology, The Chinese University of Hong Kong, Hong Kong, Hong Kong SAR, China.; ^6^ Centre for Robotics and Automation, City University of Hong Kong Shenzhen Research Institute, Shenzhen 518057, China.

## Abstract

Mitochondrial transfer is a spontaneous process to restore damaged cells in various pathological conditions. The transfer of mitochondria to cell therapy products before their administration can enhance therapeutic outcomes. However, the low efficiency of previously reported methods limits their clinical application. Here, we developed a droplet microfluidics–based mitochondrial transfer technique that can achieve high-efficiency and high-throughput quantitative mitochondrial transfer to single cells. Because mitochondria are essential for muscles, myoblast cells and a muscle injury model were used as a proof-of-concept model to evaluate the proposed technique. In vitro and in vivo experiments demonstrated that C2C12 cells with 31 transferred mitochondria had significant improvements in cellular functions compared to those with 0, 8, and 14 transferred mitochondria and also had better therapeutic effects on muscle regeneration. The proposed technique can considerably promote the clinical application of mitochondrial transfer, with optimized cell function improvements, for the cell therapy of mitochondria-related diseases.

## Teaser

In vitro and in vivo experiments with muscle injury model demonstrated the efficiency of our mitochondrial transfer technique.

## INTRODUCTION

Heteroplasmy, which is the ratio of mutant to wild-type mitochondria DNA (mtDNA), determines the severity of mitochondria-related disorders (*1*, *2*). In muscle tissues, when heteroplasmy exceeds a certain level or the mitochondria become dysfunctional, less adenosine triphosphate (ATP) and excessive levels of reactive oxygen species are produced (*3*, *4*), which can trigger muscle atrophy, weakness, and loss of endurance (*5*, *6*). Previous clinical and preclinical animal studies demonstrated links between increased mitochondrial damage and poor skeletal muscle health (*7*, *8*). Since the 1990s, cell therapy, particularly myoblast transplantation, has been proposed to improve the regeneration of injured skeletal muscles. However, failure of early clinical trials with myoblast transplantation has been observed (*9*). Therefore, new approaches, such as macrophage regulation (*10*) and chemical induction of stem cells (*11*), have been proposed to augment muscle regeneration in skeletal muscle disorders. Restoring or improving mitochondrial functions to facilitate muscle regeneration is an attractive research focus (*8*).

Apart from energy production for cells, mitochondria can influence cell proliferation, aging, apoptosis, innate immunity, calcium homeostasis, and even stem cell differentiation potential (*12*–*14*). Mutations of mtDNA impair the functions of cells and tissues. It is evidenced that spontaneous transfer of mitochondria can occur in nature between healthy and damaged cells via different mechanisms, which is believed to protect the damaged cells and restore their cellular function (*15*). Mitochondrial transfer is a technique to alter the mtDNA in cells, and it has attracted increasing attention since it was first reported by Clark and Shay (*16*). Mitochondrial transfer has already been used in cell therapy for mtDNA-related diseases (*17*–*20*). It is easier to perform and practically more efficient (*1*) than other techniques modifying the mitochondrial genome, such as mitochondria-targeted zinc-finger nucleases (mitoZFNs) (*21*) and mitochondria-targeted transcription activator-like effector nucleases (mitoTALENs) (*22*). Transferring exogenous mitochondria into recipient cells could also reduce the ratio of mutant to wild-type mtDNA and promote the restoration or improvement of cell and tissue functions (*1*, *14*, *16*, *23*–*26*). Previous studies showed that exogenous isolated mitochondria could be delivered into cells via coculture (*16*, *24*) or microinjection (*23*, *26*).

In the method of coculture with isolated mitochondria, the isolated mitochondria are engulfed by the recipient cells via endocytosis (*16*, *24*), which is a type of cellular activity to take up objects ranging in size from nanometers to several micrometers from the surrounding environment (*27*). The isolated mitochondria randomly move around the recipient cell and have chances to be engulfed by the cell when contacting it; this phenomenon could be regarded as a random and sporadic process (*28*, *29*). The transfer efficiency of the coculture method is influenced by the quantity of extracellular isolated mitochondria. Although it reached a maximum of 28% in a previous study (*24*), the number of transferred mitochondria was considerably heterogeneous (1 to 60 mitochondria per recipient cell) even when subjected to an equal amount of isolated mitochondria (*14*). Despite being a rather simple process, the success of the coculture method is dependent on many uncontrollable factors, which might determine the unsatisfactory cell metabolism recovery rate of recipient cells in a previous work (~0.2%) (*16*). An automated optical tweezers (OT)–based manipulation system was used for qualitative and quantitative mitochondrial transfer (*14*) to reduce the influence from uncontrollable factors. The OT-based system could precisely pick up the healthy mitochondria and transport them to the target recipient cell. However, this method has a low throughput, which hinders its use for clinical applications.

Unlike coculture methods, microinjection injects isolated mitochondria preloaded in a microneedle directly into the recipient cells; thus, it may cause damage to the recipient cell owing to cell membrane opening during the delivery process (*23*, *26*). In addition, the throughput of the microinjection technique is low. In the work of King and Attardi (*23*), only 600 to 1700 cells were injected in every experiment. A photothermal nanoblade, which is a modified microneedle that can deliver small or large objects into cells more effectively, was developed later to increase the injection rate to 100 cells per hour (*26*). The use of a photothermal nanoblade increased the cell metabolism recovery rate of recipient cells to 2%, 10-fold higher than that in the work reported by King and Attardi (*23*). In 2020, Patananan’s group (*25*) proposed a modified microinjection technique, named MitoPunch, which increased the injection rate to 2 × 10^5^ cells in one experiment and exhibited higher recovery rate than those of coculture methods. However, cell membrane opening remains inevitable in the MitoPunch technique, which is not favorable to the viability of recipient cells.

All the aforementioned methods provide useful tools for studying the mechanism of cell function restoration or improvement upon mitochondrial transfer. However, they still cannot fulfill the demand of a large quantity of mitochondria transferred cell in the cell therapy industry. The coculture technique shows considerable advantage because it is harmless and has high throughput, but its low efficiency and heterogeneity are still major limitations.

Droplet microfluidics is a technology that disperses a continuous flow carrying chemical reagents, cells, or other biomaterials into discrete volumes at the micrometer scale, called droplets (*30*, *31*). These droplets are the basic unit for further chemical reactions, cell life activities, target detections, and material synthesis (*32*–*36*). Droplet microfluidics provides a considerably smaller and constrained environment than the bulk volume method, thus allowing more rapid reaction and detection of molecules/particles and interactions with encapsulated cells (*30*). Flow-focusing, T-junction, and coflow structures are the main structures used to generate droplets in current droplet microfluidic devices (*31*). Previous works have demonstrated that the droplet generation rate could be as high as thousands of droplets per second with these structures (*31*, *37*), making droplet microfluidics a high-throughput technique intrinsically. An important application of droplet microfluidics is single-cell analysis (*38*, *39*), in which a single cell is encapsulated in one droplet for analyzing cell life activities or cell modification, such as antibody analysis or gene editing (*34*, *40*–*42*). In the context of single-cell analysis applications, the single-cell encapsulation ratio, which is the ratio of the number of droplets containing one cell to the number of total droplets, is an important indicator to show how likely the interaction between encapsulated cells is in each droplet (*43*). In previous studies, using only the aforementioned droplet generation structures, the single-cell encapsulation efficiency followed a Poisson distribution, reaching approximately 33% at a relatively low multiple-cell encapsulation rate of approximately 10% at an average of 0.6 cells per droplet. Although droplets containing single cells can be sorted (*30*), adding a droplet sorting module would considerably increase the system complexity and cost of chip fabrication.

To overcome these challenges, we propose a quantitative high-throughput and high-efficiency method for mitochondrial transfer based on droplet microfluidics, which combines the advantages of coculture and droplet microfluidics. A wave-like structure was used to increase the single-cell encapsulation ratio while reducing the multiple-cell encapsulation ratio. The number of mitochondria transferred into the cells encapsulated in droplets can be controlled by adjusting the concentration of the isolated mitochondria suspension used, thus allowing quantitative control of the mitochondria transferred into recipient cells. The effectiveness of the novel mitochondrial transfer system was determined by in vitro and in vivo models of muscle cell differentiation and regeneration.

## RESULTS

### Droplet microfluidics–based mitochondrial transfer system

**Fig. 1.**
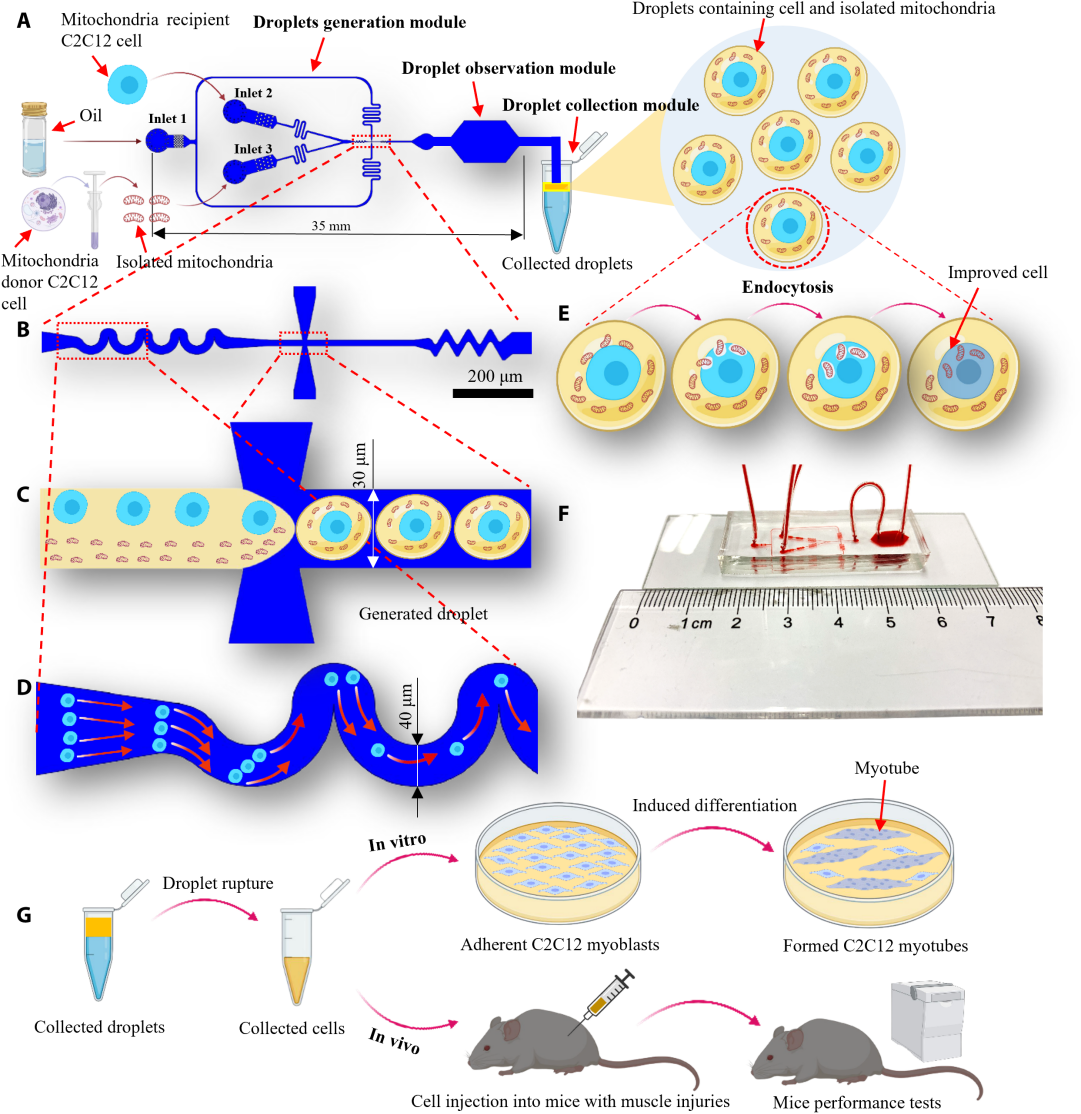
Schematic of the system setup, workflow of the droplet microfluidics–based mitochondrial transfer technique, and experimental assessments. (**A**) System setup for the droplet microfluidics–based mitochondrial transfer technique. (**B**) Wave-like structure for cell pairing before encapsulation and wave-like structure for mitochondria and cell suspension mixing after encapsulation (*56*, *57*). (**C**) Coencapsulation of cells and mitochondria in droplets. (**D**) Demonstration of cell focusing in the wave-like structure. (**E**) Demonstration of mitochondrial transfer process via endocytosis in the droplet. (**F**) Fabricated chip for droplet generation and observation. (**G**) Experimental workflow to demonstrate the effectiveness of mitochondrial transfer recipient cells on myogenic differentiation in vitro and quality and functional outcome of muscle regeneration in vivo.

The developed droplet-based mitochondria transfer system consists of three modules, namely, droplet generation, droplet observation, and droplet collection module (Fig. 1A). We loaded the mitochondria-recipient C2C12 cell suspension, isolated mitochondria suspension, and surfactant-added fluorinated oil (Sphere Fluidics, C021) into the droplet generation module via three inlets. Then, we collected the generated droplets in the collection tube (fig. S1E). The flow-focusing structure (Fig. 1, B and C) was used to separate the two suspensions into droplets (*37*, *44*). A wave-like structure before the flow-focusing structure (droplet generation position) was used to align randomly distributed cells from the inlet to a line to improve the single-cell encapsulation efficiency (Fig. 1D; fig. S1, A to D; and movies S1 and S2). Another wave-like structure behind the flow-focusing structure was used to mix the mitochondria and cell suspensions within the droplets (Fig. 1B). The isolated mitochondria were taken up by the recipient C2C12 cells via endocytosis inside the droplets (Fig. 1E). The droplet generation and observation modules were connected via a polyethylene tubing (BD Intramedic, BD 427406), and the entire fabricated chip was only approximately 8 cm in length (Fig. 1F). After the mitochondrial transfer was completed, the recipient cells were collected from the droplets via a droplet rupture process (Materials and Methods), and functional experiments could be immediately performed to assess the effect of mitochondrial transfer at different quantities on C2C12 cell myogenic differentiation in vitro and muscle regeneration in vivo (Fig. 1G).

**Fig. 2.**
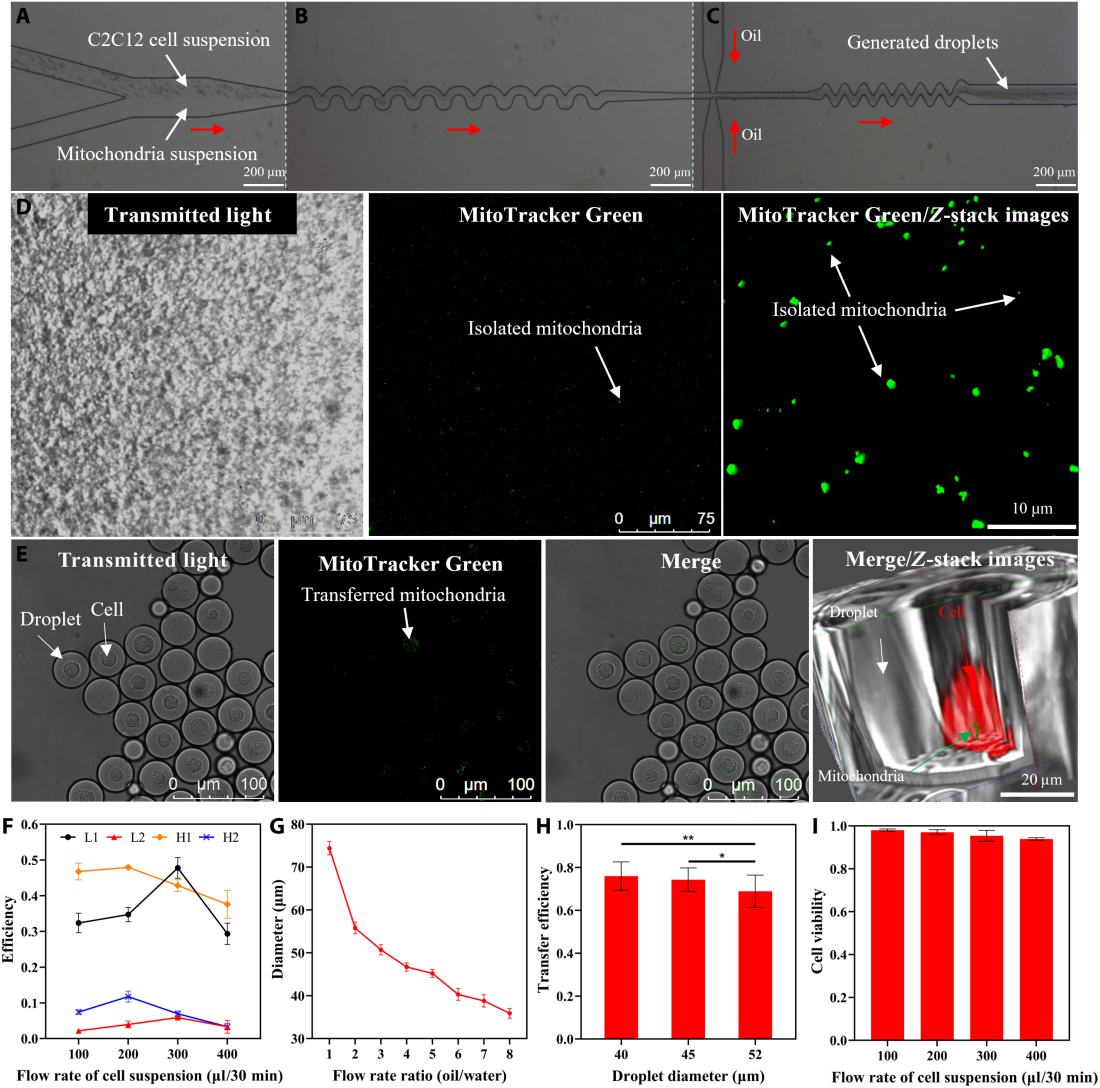
Presentation of droplet microfluidics–based mitochondrial transfer system. (**A**) Coflow of cell and isolated mitochondria suspensions to the wave-like structure for cell focusing. (**B**) Wave-like structure used for cell focusing to improve single-cell encapsulation efficiency (more details in fig. S1). (**C**) Flow-focusing structure for droplet generation and wave-like structure for mixing of cell and mitochondria suspensions within the generated droplets. (**D**) MitoTracker Green FM–stained mitochondria were isolated from C2C12 myoblasts and observed under confocal microscope for 2D and 3D *Z*-stack images. (**E**) An aliquot portion of droplets were collected for further confocal imaging analysis to verify the extent of mitochondrial transfer by counting the number of transferred mitochondria prestained with MitoTracker Green FM. The recipient cell membrane was stained with CellMask Deep Red. Representative 3D image of a droplet (gray) was segmented to visualize the encapsulated mitochondria (green) inside a recipient cell (red). (**F**) Cell encapsulation efficiency using the wave-like structure under different concentrations of cell suspension and different flow rates. Here, 1 and 2 stand for one and two or more cells encapsulated in one droplet, respectively. L stands for 0.85 × 10^7^ cells/ml, and H stands for 1.7 × 10^7^ cells/ml. (**G**) Influence of oil/water flow rate ratio on droplet size. (**H**) Influence of droplet size on mitochondrial transfer efficiency. (**I**) Influence of cell suspension flow rate on cell viability. All images from (D and E) were acquired with a confocal microscope (see Materials and Methods for details). All data from (F to I) were acquired from three independent experiments, presented as means ± SD, and analyzed by one-way analysis of variance (ANOVA) with Dunn’s multiple comparisons test. *P < 0.05 and **P < 0.01. The red arrows from (A to C) indicate the flow directions inside channels.

For observation and three-dimensional (3D) rebuilding under a confocal fluorescence microscope (Leica SP8LIA++ True Confocal Laser Scanning Microscope), we used MitoTracker Green FM (Invitrogen, M7514) and CellMask Deep Red (Invitrogen, C10046) to stain the mitochondria (before isolation from donor cells) and recipient cells, respectively. After mitochondria isolation, we immediately loaded the mitochondria-recipient cells and freshly isolated mitochondria into the microfluidic chip for the encapsulation and mitochondrial transfer process (Fig. 2, A to C). The labeled isolated mitochondria in suspension were spherical-like structures with a diameter of ~1 μm (Fig. 2D). After 2 hours of coculture in the generated droplets, the isolated mitochondria were taken up by the recipient cells via endocytosis (Fig. 2E). As mentioned above, the cell encapsulation efficiency was improved beyond the Poisson distribution by using the wave-like structure. The cell aligning effect was affected by flow velocity within the wave-like structure (*45*). At a cell concentration of 0.85 × 10^7^ cells/ml and a cell suspension flow rate of 300 μl per 30 min (the flow rate of the isolated mitochondria suspension was the same), the wave-like structure showed better cell focusing, indicating less cell aggregation and a more suitable distance between two adjacent cells, compared to other flow rate and cell suspension concentration measurements (fig. S1C). Furthermore, the single-cell encapsulation efficiency could reach approximately 47.8%, whereas the multiple-cell encapsulation ratio was suppressed to approximately 5.9% (Fig. 2F). This implies that the single-to-multiple cell encapsulation ratio was increased to 8.1, in other words, 292% of the Poisson distribution (λ = 0.6; fig. S1B). Such improvement in single-cell encapsulation efficiency helps decrease the proportion of undesired droplets in the produced droplets and increase the throughput. The proposed system could notably yield 2 × 10^6^ recipient cells in droplets for mitochondria transfer in 30 min. The mitochondrial transfer efficiency, defined as the ratio of the isolated mitochondria transferred into the cell to the total isolated mitochondria encapsulated in the droplet, decreased slightly from 75 to 70% with the increase in droplet diameter from 40 to 52 μm (Fig. 2H). In this work, the droplet diameter could be set to 40 μm by simply setting the flow rate ratio of the oil/water phase to 6 (Fig. 2G) (*44*) given that the properties of the encapsulated cell do not affect the size of the generated droplets (*30*). After processing with the developed system, the viability of the recipient cells could still be kept relatively high (e.g., 95% at a flow rate of 300 μl per 30 min; Fig. 2I).

### High-efficiency quantitative mitochondrial transfer

**Fig. 3.**
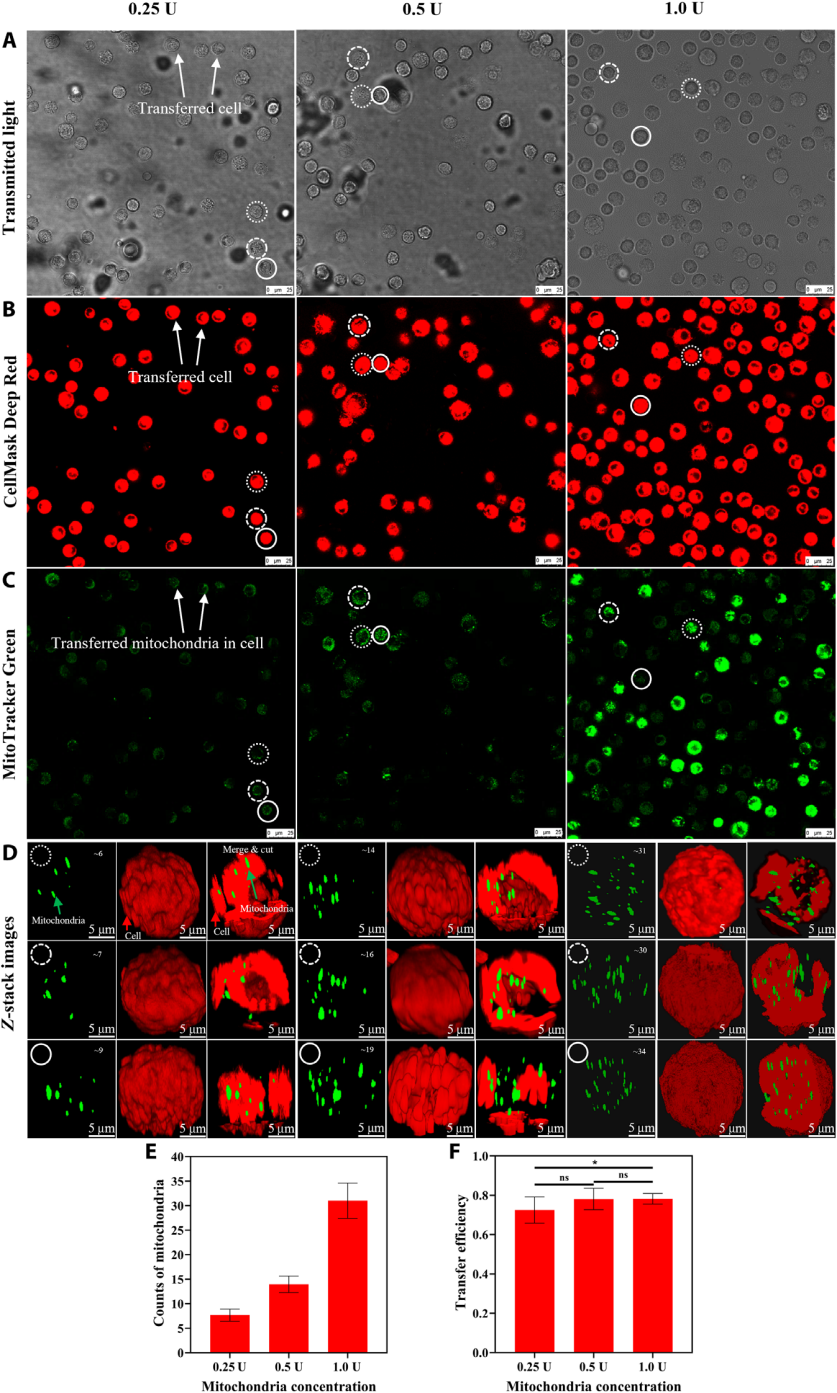
Quantitative control of mitochondrial transfer using the proposed droplet–based method. (**A** and **B**) Mitochondrial transfer recipient cells (using 0.25, 0.5, and 1.0 U of mitochondria suspensions, where 1.0 U of mitochondria stands for the concentration of mitochondria isolated from 1 × 10^6^ cells and suspended in 10 μl mitochondria storing reagent) observed under the confocal microscope. The cell membranes of recipient cells were stained with CellMask Deep Red (excitation/emission: 633/655 nm). (**C**) The presence of transferred mitochondria inside recipient cells was visualized using MitoTracker Green FM (excitation/emission: 488/512 nm); mitochondria were stained before isolation and transfer. (**D**) Representative 3D *Z*-stack images reconstructed from 2D confocal images of the mitochondrial transfer recipient cell in (A and B). The 3D images of recipient cells (red) were segmented to visualize the transferred mitochondria (green). White circles denote the selected recipient cells in low magnification. (**E**) Average number of transferred mitochondria per cell under different mitochondria concentrations. The number of mitochondria was counted in the 3D images using a confocal fluorescence microscope. (**F**) Transfer efficiency calculated as the ratio of the number of isolated mitochondria transferred into a recipient cell to the total number of isolated mitochondria encapsulated in a droplet. All images from (A) were acquired with a confocal microscope (see Materials and Methods for details). All data in (E and F) were acquired from three independent experiments, presented as means ± SD, and analyzed by one-way ANOVA with Dunn’s multiple comparisons test. *P < 0.05. ns, not significant.

The closed microenvironment of droplets limited the traveling distance of isolated mitochondria and increased the probability of the isolated mitochondria to contact the cell (movie S3), thereby facilitating the uptake of mitochondria by the cell and improving the mitochondria transfer efficiency. Moreover, because of the smaller size of isolated mitochondria compared to that of the droplets (1 to 40 μm in diameter), the isolated mitochondria were evenly encapsulated in each droplet. The number of isolated mitochondria encapsulated in droplets could be controlled by adjusting the concentration of the isolated mitochondria suspension (fig. S2). In this study, three different concentrations of isolated mitochondria suspension were used to verify the transfer efficiency of the proposed system. Here, 1.0 U of concentration refers to mitochondria isolated from 1 × 10^6^ cells and suspended in 10 μl of mitochondrial storing reagent (Beyotime, C3601-3). Using 0.25, 0.5, and 1.0 U of concentration, 8, 14, and 31 isolated mitochondria were transferred into the recipient cells on average, respectively (Fig. 3, A to E). The mitochondrial transfer efficiency slightly increased from 73 to 78%, with the concentration increasing from 0.25 to 1.0 U (Fig. 3F). The time spent for the transfer affects the transfer efficiency. Given the loss of mitochondrial viability after isolation (*46*), the transfer time was set to 2 hours. If the transfer time could be longer, then the difference in transfer efficiency among the three cases would be more evident.

### In vitro study of mitochondrial transfer effect on promoting proliferation and myogenic differentiation of C2C12 cells

**Fig. 4.**
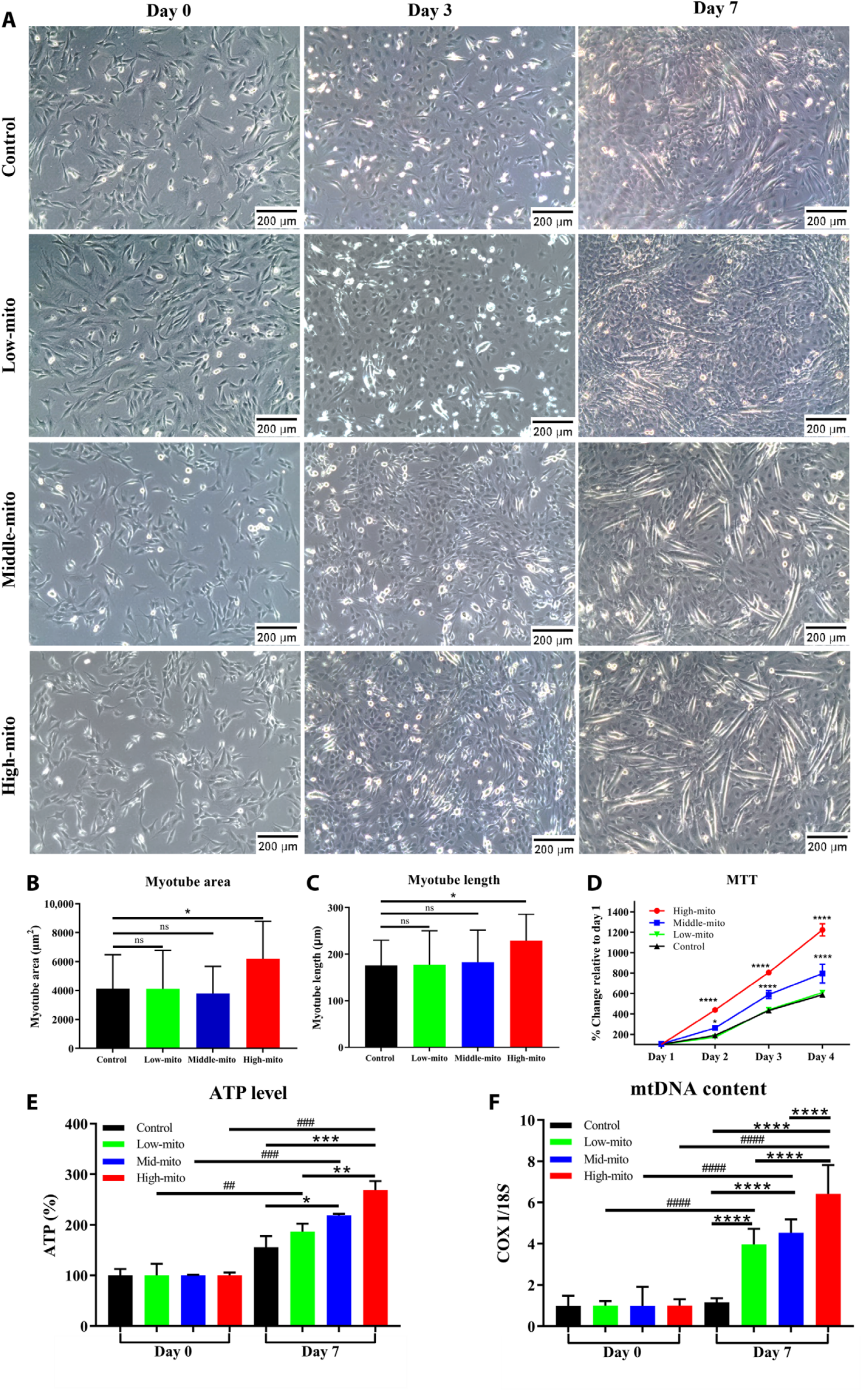
In vitro study of mitochondrial transfer effect on myogenic differentiation of C2C12 myoblast cells. (**A**) Representative bright-field images of mitochondria-transferred C2C12 cells during the myogenic induction process. C2C12 cells were subjected to mitochondrial transfer at different concentrations before myogenic induction (8, 14, and 31 exogenous isolated mitochondria transferred per cell were defined as low-mito, mid-mito, and high-mito transferred groups, respectively). The cell morphology and formed myotubes were imaged right before the induction started and on days 3 and 7 of the induction process. (**B** and **C**) On day 7, the myotube area and length were determined using ImageJ, and three field of views were taken per well. (**D**) Cellular proliferation of C2C12 cells was determined by MTT assay on days 1 to 4 after mitochondrial transfer. (**E** and **F**) Intracellular ATP levels and mtDNA content of C2C12 cells were quantified on days 0 and 7 after mitochondrial transfer. ATP was measured with the ATP Colorimetric Assay Kit, while mtDNA content was indicated by the ratio of mtDNA’s cytochrome c oxidase subunit I (Cox1) gene to 18*S* nuclear DNA (mtDNA/nDNA) with qPCR. All the values were normalized to day 0. Data were acquired from three independent experiments, presented as means ± SD, and analyzed with one-way ANOVA followed by Dunn’s multiple comparison test. *P < 0.05 (or #), **P < 0.01 (or ##), ***P < 0.001 (or ###), and ****P < 0.0001 (or ####).

To test the differentiation ability of C2C12 myoblasts after mitochondrial transfer using the proposed droplet microfluidics–based method, we performed myogenesis assays. After 7 days of induction, the myotube area and length (as indicators of myogenesis) showed a significant increase in the high-mito transferred group (31 exogenous isolated mitochondria transferred per cell) compared with the control, low-mito transferred, and mid-mito transferred groups (corresponding to 0, 8, and 14 exogenous isolated mitochondria transferred per cell, respectively; Fig. 4, A to C). Moreover, the 3-(4,5-dimethylthiazolyl-2)-2,5-diphenyltetrazolium bromide (MTT) assay indicated 2.5 and 1.5 times increase in cell proliferation on day 4 in the high- and mid-mito transferred groups, respectively (Fig. 4D). The ATP level and mtDNA content of the high-mito transferred group showed a remarkable increase on day 7 compared with that of the other groups, and this finding was correlated with the number of transferred mitochondria (Fig. 4, E and F).

**Fig. 5.**
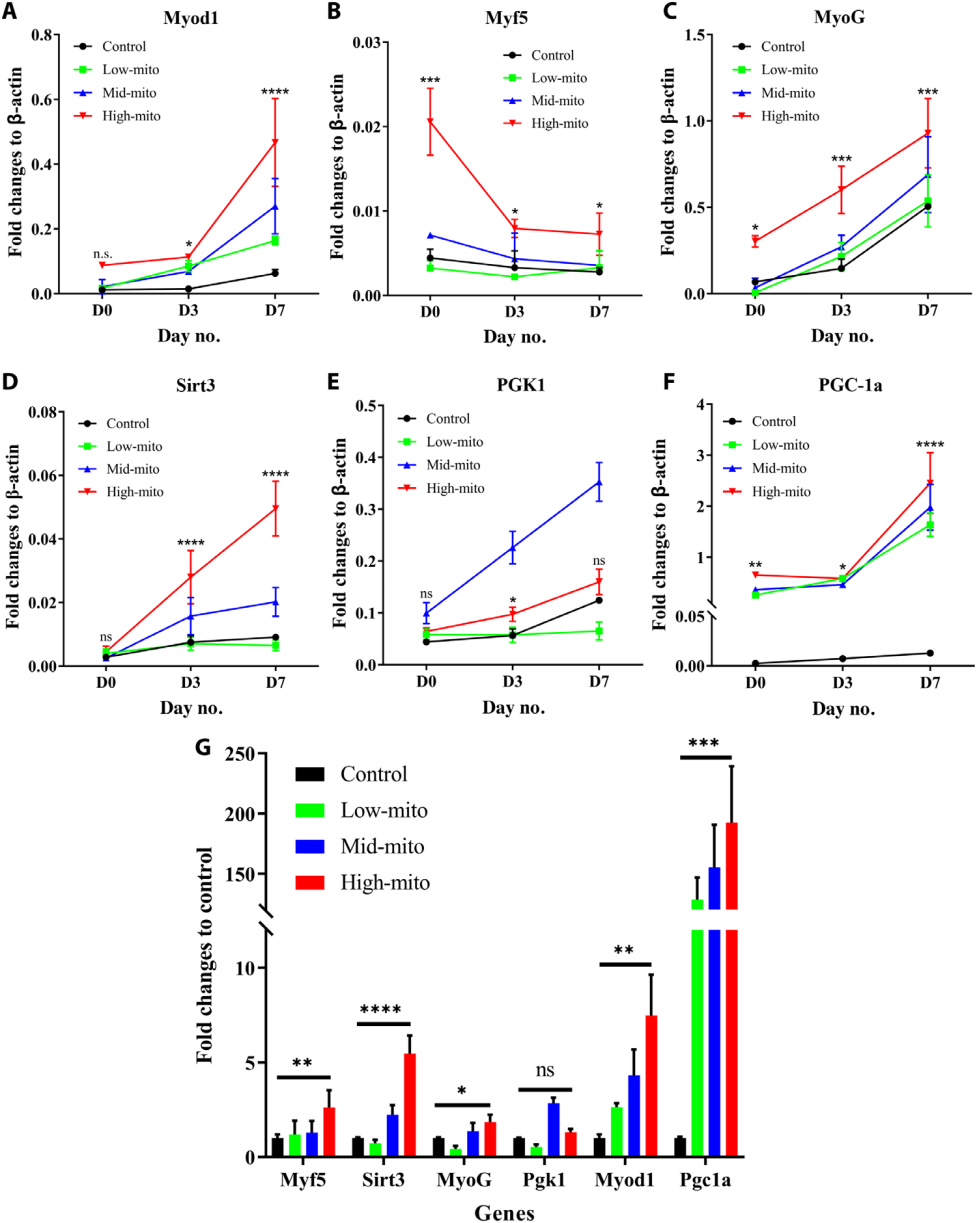
Expression of myogenic and mitochondria-related genes during myogenic differentiation in C2C12 cells after mitochondrial transfer. (**A** to **C**) Expression levels of muscle regeneration–related genes. (**D** to **F**) Expression levels of mitochondria-related genes. (**G**) mRNA expression level in different treatment groups and control group at day 7. The expression level was presented as fold change with respect to the control group. All data were acquired from three independent experiments, presented as means ± SD, and analyzed with one-way ANOVA followed by Dunn’s multiple comparisons test. *P < 0.05, **P < 0.01, ***P < 0.001, and ****P < 0. 0001.

Furthermore, to test the expression of myogenic markers (Myod1, Myf5, and MyoG) and mitochondria-related markers [sirtuin 3 (Sirt3), phosphoglycerate kinase 1 (Pgk1), and peroxisome proliferator-activated receptor gamma coactivator 1-alpha (Pgc-1a)] at the molecular level, we performed a quantitative polymerase chain reaction (qPCR) analysis. All other markers except for Pgk1 showed up-regulated expression upon mitochondrial transfer in a concentration-dependent manner from day 0 to 7 (Fig. 5, A to F). In particular, after 7 days of induction, all the tested markers except for Pgk1 of the high-mito transferred C2C12 myoblasts still showed significantly up-regulated expression compared with the control group (Fig. 5G). It was remarkable that the Pgk1 of the mid-mito transferred group showed significant up-regulation compared with that of the other groups, which may be due to the fact that too few or too many mitochondria transferred could not promote the expression of Pgk1, as found in a previous work (*14*). This phenomenon further indicated the importance of quantitative control of mitochondrial transfer in precise medicine.

The above in vitro experimental results showed that mitochondria transfer at a number of 31 per cell could significantly enhance the myogenic differentiation and ATP production ability. On the basis of these results, we used high-mito transferred cells in the subsequent animal experiments to study the functional outcome and dose effect of the cell therapy treatment.

### Intramuscular injection of mitochondria-transferred C2C12 to improve muscle healing quality and functional outcome

**Fig. 6.**
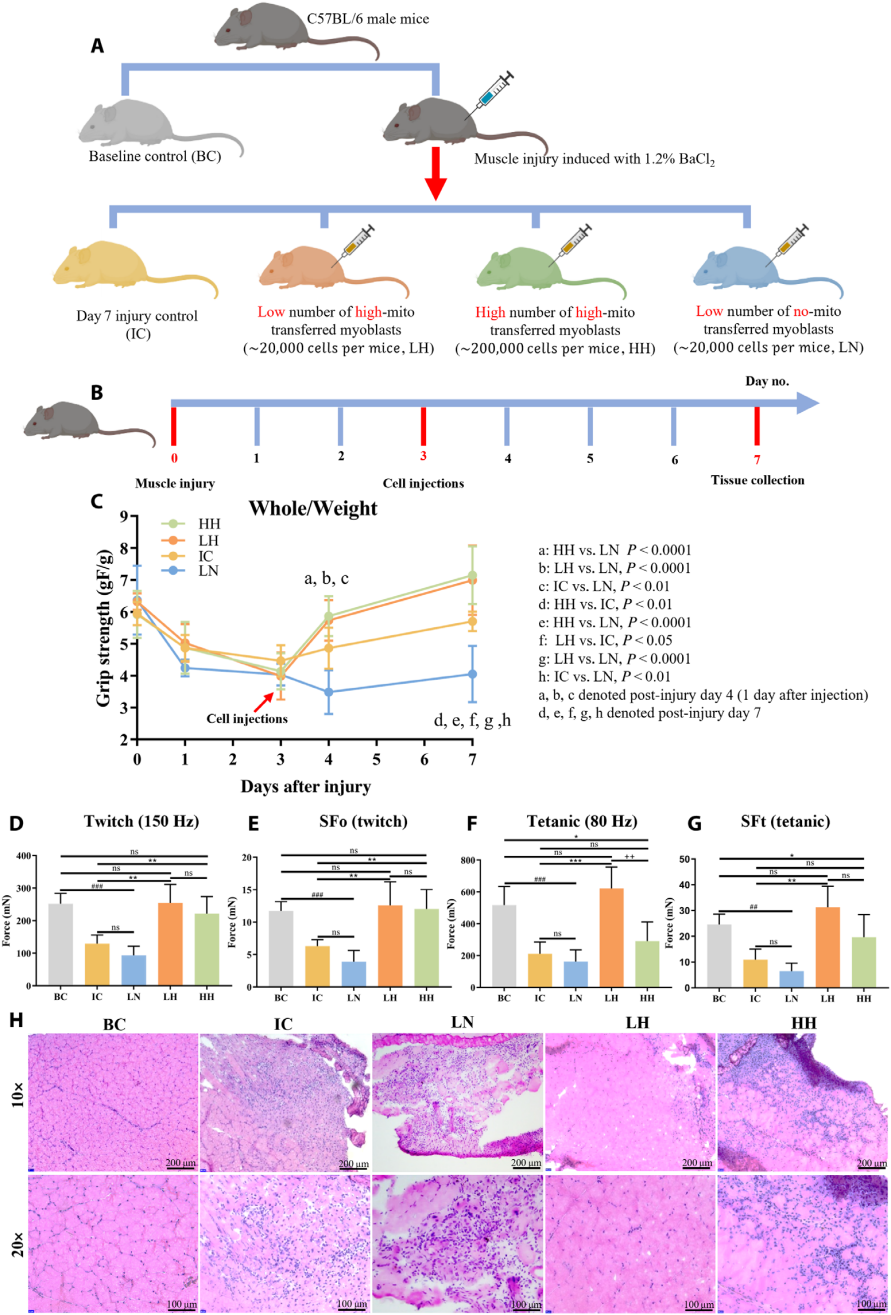
In vivo study of mitochondria-transferred C2C12 injection effect on muscle regeneration. (**A**) Five groups of C57BL/6 mice were used to study the therapeutic effects (Materials and Methods). (**B**) Timeline of the mouse treatments. Muscle injuries were induced with 1.2% BaCl_2_ on day 0, followed by cell injections on day 3. Gastrocnemius muscle tissue from all five groups was collected on day 7. (**C**) The whole-body grip strengths of IC, LN, LH, and HH groups were tested and normalized to the mouse body weight. LH and HH groups showed larger whole-body grip strength than IC and LN groups from days 4 to 7. (**D** to **G**) Fo, SFo, Ft, and SFt of all five groups on day 7. LH and HH groups were recovered to the BC group, whereas IC and LN groups still showed performance defects. Force measurements were performed ex vivo on gastrocnemius muscle from anesthetized mice. (**H**) H&E staining of gastrocnemius muscle tissue from all five groups. LH and HH groups showed regenerated myofibers undergoing progressive growth and maturation, highlighted by the increasing cross-sectional area and nuclear relocation toward the periphery. All data were presented as means ± SD and analyzed with two-way ANOVA followed by Tukey’s post hoc test (*n* = 5). *P < 0.05 (# or +), **P < 0.01 (## or ++), ***P < 0.001 (### or +++), and ****P < 0.0001 (#### or ++++).

We performed in vivo study on mice, and randomly and evenly divided 25 C57BL/6 male mice (3 months old; 25 to 30 g) into five groups with different treatments (Materials and Methods and Fig. 6A). Muscle injury was induced by BaCl_2_ on day 0, followed by cell injections on day 3 (Fig. 6B). On days 4 and 7, the whole-body grip strength of mice receiving a low dose (20,000 cells per mouse) of high-mito transferred myoblasts (LH) or a high dose (200,000 cells per mouse) of high-mito transferred myoblasts (HH) was significantly improved (Fig. 6C). Compared with those in the injury control (IC; without intervention) and low number (20,000 cells per mouse) of no-mito transferred myoblast (LN) groups, the twitch force (Fo), specific Fo (SFo), tetanic force (Ft), and specific Ft (SFt) were all significantly increased in the LH and HH groups [Materials and Methods and Fig. 6 (D to G)].

Compared with baseline control (BC), the hematoxylin and eosin (H&E)–stained cryosection of muscle tissues collected from the IC and LN groups showed clear evidence of muscle damage with muscle fiber disintegration and centrally located nuclei, whereas the muscle tissues from the LH and HH groups were characterized by regenerated myofibers undergoing progressive maturation, as indicated by the increasing cross-sectional area and nuclear relocation toward the periphery (Fig. 6H). The difference between LN and LH clearly indicated the benefit of mitochondrial transfer on cell-mediated muscle regeneration. Moreover, cell infiltration was observed in the HH group, suggesting the likelihood of immune response upon injection of the high cell dose, whereas the low-dose injection of mitochondria-transferred C2C12 cells led to the most satisfactory healing outcome in this study. A notable detail was that the LN and IC groups had no significant difference with each other in all the tests except for the whole-body grip strength. With these data, speculating that myoblast transplantation without prior mitochondrial transfer is not sufficient to promote the healing process of injured muscles is reasonable and consistent with previous findings (*9*, *47*).

**Fig. 7.**
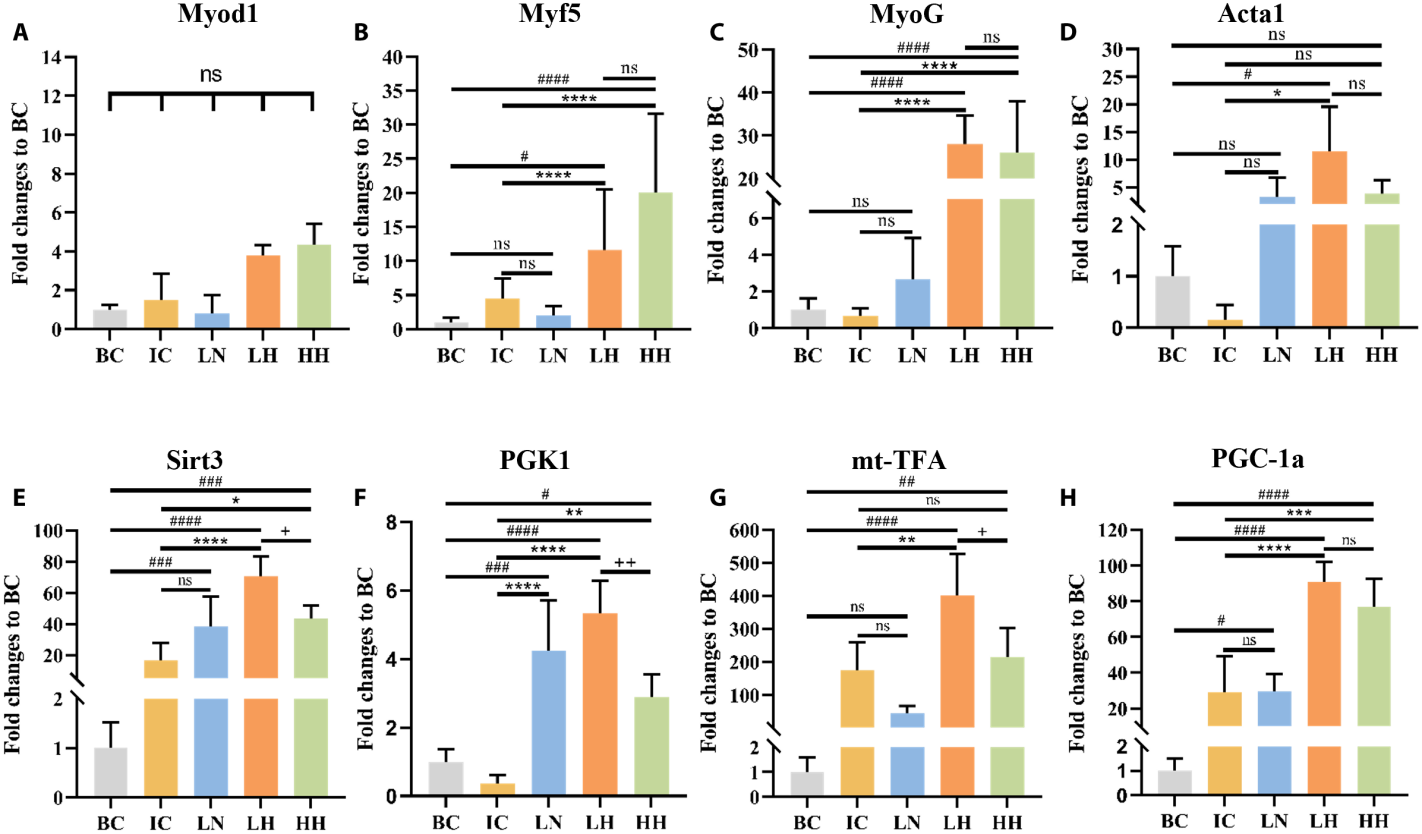
Muscle myogenic and mitochondria metabolic gene expression levels of muscle tissue–injured mice after C2C12 cell injections. (**A** to **C**) Expression levels of muscle regeneration–related genes. (**D**) Expression levels of structural protein Acta1 gene. (**E** to **H**) Expression levels of mitochondria metabolic genes. All data were presented as means ± SD and analyzed with two-way ANOVA followed by Tukey’s post hoc test. *P < 0.05 (# or +), **P < 0.01 (## or ++), ***P < 0.001 (### or +++), and ****P < 0.0001 (#### or ++++).

After muscle tissues were collected from all five groups, we performed RNA extractions and a qPCR analysis (Materials and Methods). Compared with those of the IC and LN groups, the mRNA levels of myogenic markers (Myf5 and MyoG), skeletal muscle α-actin (Acta1), and mitochondrial genes (Sirt3, Pgk1, mitochondrial transcription factor A, and Pgc-1a) showed significant up-regulation in muscle tissues after intramuscular injection of mitochondria-transferred C2C12 cells (LH and HH groups; Fig. 7). In general, the highest up-regulation was found in LH, which is consistent with the optimal healing outcome in functional assessment. Except for Pgk1, injection of C2C12 without prior mitochondrial transfer (LN) did not induce gene expression change compared with the IC group.

## DISCUSSION

In the present study, we developed a novel droplet microfluidics–based mitochondrial transfer method to overcome the limitation of quantitative high-throughput transfer. In particular, exogenous isolated mitochondria and a single recipient cell were encapsulated in one droplet, and then, the isolated mitochondria were taken up by the recipient cell via endocytosis. As the droplet provides a closed environment, the space allowed for the movement of isolated mitochondria is considerably smaller than the open environment in traditional coculture methods. Consequently, the probability of mitochondria uptake within droplets is significantly increased. To further improve the system performance, we used a wave-like structure to align the randomly distributed cells from the inlet into a linear flow, which helped increase the single-cell encapsulation ratio to ~47.8% while reducing the multicell encapsulation ratio to ~5.9% at an average of 0.6 cells per droplet. Compared with the droplet sorting module, the wave-like structure was easy to integrate into the droplet microfluidic chip without increasing the complexity and cost of the system.

Furthermore, the optimization of the shape of the wave-like structure is one of the future efforts to further improve the performance of the system. The number of mitochondria transferred into recipient cells can be controlled by adjusting the concentration of the isolated mitochondria suspension used. Compared with the traditional coculture method (*16*, *24*) and the recently proposed OT-based method (*14*), the method proposed in the present study can achieve quantitative control of mitochondrial transfer into recipient cells at the single-cell level while maintaining a high mitochondria transfer efficiency of 75% and a high throughput, generating 2 × 10^6^ mitochondria-transferred cells in 2.5 hours (0.5 hours for generating droplets containing the cells and isolated mitochondria and 2 hours for the mitochondrial transfer process in droplets). The previous work showed that the number of mitochondria transferred into recipient cells increased with the time used for the transfer process. We limited the mitochondrial transfer process to 2 hours, which resulted in an incomplete transfer process. If we could extend the transfer time without losing isolated mitochondria viability (*46*), then more isolated mitochondria could be taken up by recipient cells and the transfer efficiency could be further increased. By using such a new technology, a large number of viable C2C12 cells with mitochondrial transfer at different settings were generated for subsequent in vitro and in vivo functional experiments. It can be estimated that by using multiple production pipelines in an industry setting, clinical needs can be addressed without compromising time and cell quality.

The method reported in this study could allow control of the number of mitochondria transferred into recipient cells, thus enabling the study of the dose effect of mitochondrial transfer on muscle regeneration with high throughput. In vitro studies including myoblast proliferation rate, differentiation ability, ATP production, and mtDNA content analysis revealed that transferring more mitochondria into C2C12 myoblasts could lead to increased cell proliferation rate and myotube formations. Moreover, in vivo studies on mice, including ex vivo functional tests on gastrocnemius, histology, and qPCR analyses, indicated that direct intramuscular injection of low dose of high-mito transferred myoblasts resulted in the most satisfactory outcome of skeletal muscle regeneration. Transplantation of proliferative myoblasts has been proposed to mitigate progressive reduction in muscle mass and strength due to genetic disorders or aging. However, the efficacy of previous clinical trials is not satisfactory because of several potential factors such as rapid cell death after transplantation and graft rejection (*48*, *49*). The findings in in vitro and in vivo studies provided evidence that quantitative controlled high-throughput mitochondrial transfer could improve the regeneration outcome of cell therapy. Further investigation is necessary to examine the cellular response upon mitochondrial transfer at the single-cell level and the efficacy and immune response in longer treatment duration.

The importance of the mitochondria in energy production, signal transduction, and the aging process has been well recognized. Apart from regulating the stem cell proliferation and differentiation, the implication of mitochondrial transfer has been an intriguing observation in cell therapy. Stem cells could transfer their mitochondria to recipient cells under stress, partly contributing to the therapeutic effect of stem cell therapy in various conditions including brain ischemia and smoke-induced lung damage (*50*, *51*). The benefit of mitochondrial transfer from healthy cells to stressed cells on restoring the metabolic function was recently demonstrated in osteocytes via dendritic network (*20*). However, controlling the efficiency of cell-mediated mitochondrial transfer in vivo is technically difficult. By contrast, the present study demonstrated that enhancing the effectiveness of cell therapy with mitochondrial transfer before administration is feasible. The findings are clinically important because genetic, morphological, and phenotypic changes in cell therapy products upon ex vivo expansion (*52*) and in stem cells isolated from aged donors (*53*) are not uncommon. The present study opens up a new avenue to manipulate cell therapy products using a high-efficiency and high-throughput transfer technique before application, which has been previously hampered by a low transfer rate and low throughput.

## MATERIALS AND METHODS

### Microfluidic chip fabrication and operations

The designed chip was fabricated using soft lithography (fig. S4) (*54*). Before the experiments, the chip channels were coated with a surface modification agent [Wei Na (Wuhan) Biotechnology] to make them hydrophobic for stable water-in-oil droplet generation and transporting.

### Cell culture

C2C12 myoblasts were cultured in Dulbecco’s modified Eagle medium (DMEM) with high glucose (Gibco, 11965084) supplemented with 10% fetal bovine serum (Gibco, 12800058) and 1% antibiotic-antimycotic (Gibco, 15240096) at 37°C in 5% CO_2_.

### Mitochondria isolation

The mitochondria used in this work were freshly isolated from C2C12 myoblast cells following the protocol of the mitochondria isolation kit (Beyotime, C3601) before each mitochondrial transfer experiment. First, the mitochondria of donor C2C12 cells were stained with MitoTracker Green FM (Invitrogen, M7514). Second, the stained cells were washed three times with phosphate-buffered saline (PBS), detached from the culture flask with trypsin/EDTA solution (Gibco, R001100), and centrifuged at 500*g* for 5 min. Third, the supernatant was removed, and the collected cells were resuspended with 1 ml of cell lysis reagent (Beyotime, C3601-1) and placed in an ice bath for 15 min. Fourth, the lysed cells were homogenized with a glass homogenizer for 30 cycles. Fifth, the homogenized cells were centrifuged at 1000*g* for 10 min at 4°C. Then, the supernatant was resuspended with 1 ml of cell lysis reagent (Beyotime, C3601-1) and centrifuged at 1000*g* for 10 min at 4°C again for more purity. Last, the supernatant was collected and centrifuged at 3500*g* for 10 min at 4°C. The pellet collected was the isolated mitochondria. Mitochondria storage reagent (Beyotime, C3601-3) was used to suspend the isolated mitochondria at the required concentration for further experiments. The mitochondria isolated from 1 × 10^6^ cells and suspended in 10 ml of mitochondria storing reagent was set as 1 U of mitochondrial suspension.

### 3D reconstruction of cells and mitochondria under confocal fluorescence microscope

After coculturing the recipient cells and exogenous isolated mitochondria in droplets for 2 hours, the droplets were loaded into the observation module, as shown in Fig. 1A. A confocal fluorescence microscope (Leica SP8LIA++ True Confocal Laser Scanning Microscope) was used to take images of droplets containing single cells (labeled with CellMask Deep Red) and isolated mitochondria (labeled with MitoTracker Green FM) with the following settings: 40× water immersion lens, 1.0 Airy Unit pinhole, 512 × 512 format, and 400-Hz scanning speed. MitoTracker Green FM was excited by a 488-nm laser, and emission at 512 nm was acquired by a hybrid detector. CellMask Deep Red was excited by a 633-nm laser, and emission at 655 nm was acquired using another hybrid detector. Transmitted light images and fluorescence images were taken simultaneously under a confocal microscope. The 2D images were then used for 3D reconstruction by built-in software. The isolated mitochondria transferred into the cells were counted as the green particles inside the red fluorescence–labeled cells, and the green particles outside the red cell were counted as the untransferred isolated mitochondria. Segmentation of 3D images was conducted to visualize all the transferred mitochondria. The mitochondrial transfer efficiency was calculated as the ratio of the transferred mitochondria to the total mitochondria inside the droplet. The same process was repeated for the cells recovered from droplets to count the number of mitochondria transferred under the different concentration of isolated mitochondria.

### Droplet rupture and cell collection

The collected droplets floated on top of the fluorinated oil, as shown in fig. S1E. Before the droplets broke, the extra oil on the bottom of the tube was removed. Then, 1 ml of 50% 1*H*,1*H*,2*H*,2*H*-perfluorooctanol (Thermo Scientific, AAB2015609) was added to the tube containing the collected droplets and gently vortexed for 1 min for the droplets to merge into a bulk solution. Afterward, the upper bulk solution was carefully moved to a new tube and centrifuged at 300*g* for 3 min to collect the cells.

### Cell viability test

The viability of cells after coculturing for 2 hours in droplets was tested to examine the influence of the proposed method on cells. After coculturing for 2 hours in droplets, cells were first recovered from the droplets to be a cell suspension via the droplet rupture process. Then, the cell suspension was stained by propidium iodide staining buffer (100 μl/ml; Invitrogen, P3566). After incubation at 37°C for 5 min, 20 μl of stained cell suspension was observed under the fluorescence microscope (Nikon Eclipse Ts2R). Only dead cells showed red fluorescence. Cell viability was calculated as the ratio of the number of cells without red fluorescence to the total number of cells.

### Myogenic differentiation of C2C12

For evaluating C2C12 differentiation, 5000 cells/cm^2^ were seeded in a six-well plate and cultured in growth media until reaching 80% confluence. The media were then replaced with DMEM (Gibco, 11965084) supplemented with 2% horse serum (Gibco, 16050130). The cells were kept in differentiation medium until the end of the assay, typically between days 5 and 7. Myotube formation was monitored every 2 days. The time points were days 0, 3, and 7.

### MTT assay

The cell proliferation rate of each C2C12 cell group was determined by MTT assay. Briefly, C2C12 cells were plated at a density of 5000 cells/cm^2^ in 96-well plates and incubated for 24 hours. After incubation, the cells were treated with MTT (100 μl, 0.5 mg/ml) for 3 hours at 37°C. The produced dark blue formazan crystals were solubilized using 100 μl of dimethyl sulfoxide. The absorbance at 570 nm was measured with a microplate reader.

### Quantification of ATP level

Before the assay, the cells were deproteinized immediately with a perchloric acid deproteinization kit (BioVision, K808-200). In accordance with the manufacturer’s instruction, ATP levels were measured with an ATP colorimetric assay kit (BioVision, K354-100). The absorbance at 570 nm was measured with a microplate reader. The ATP content was lastly normalized to the protein level and expressed as a percentage of control.

### Mitochondrial DNA content

Total DNA was extracted from cells using DNAzol (Thermo Scientific, 10503027). The concentrations of total DNA were measured using a NanoDrop 1000 spectrophotometer (Thermo Scientific). Total DNA stocks were subsequently diluted in water to a final concentration of 10 ng/μl. The mtDNA content was indicated by mtDNA/nuclear DNA (nDNA) ratio with qPCR. The mtDNA’s cytochrome c oxidase subunit I (Cox1) gene and the 18*S* nDNA gene were amplified by qPCR as described below. The results were presented as a percentage of the corresponding control.

### Animal treatments

C57BL/6 male mice (3 months old; body weight of 25 to 30 g) were obtained from the Laboratory Animal Services Centre. Five mouse samples were collected without treatment (BC group). Twenty mice were intramuscularly injected with 1.2% (w/v) barium chloride to induce muscle injury on day 0. In sequence, they were randomized into four groups receiving intramuscular injection in gastrocnemius of different treatments on day 3. The four groups were (i) LH (~20,000 cells per mice), (ii) HH (~200,000 cells per mice), (iii) LN (~20,000 cells per mice), and (iv) PBS IC on day 7. Samples were harvested on day 7. Ethics approval was obtained for this animal experiment from the Ethics Committee of the Chinese University of Hong Kong (Animal Experimentation Ethics Committee reference no. 18-256-MIS).

### Whole-body grip strength test

A grip strength meter (Ugo Basile, 47200) was used to measure the whole-body grip strength. The mouse was induced to grasp the grasping grids; a digital force transducer recorded the peak tension in gram force (gf). The gauge was reset to 0 gf after stabilization. Peak tension was recorded by the gauge when the mouse released its whole-body limbs from the grids. Three measurements were performed at days 0, 1, 3, 4, and 5. Each mouse was measured three times, and the average of the three measurements was calculated. The grip strength data were normalized against the bodyweight of mice on the day of measurement.

### Muscle functional assessment

Under general anesthesia, the gastrocnemius muscle of the hindlimb was isolated and then mounted on a holder vertically to the dual-mode muscle lever arm system (300C-LR, Aurora Scientific Inc., Newmarket, Canada). The intact muscle was incubated in Ringer solution (121 mM NaCl, 5.4 mM KCl, 1.2 mM MgSO_4_.7H_2_O, 25 mM NaHCO_3_, 5 mM Hepes, 11.5 mM glucose, and 25 mM CaCl_2_) maintained at room temperature and continuously pumped with a gaseous mixture containing 95% O_2_ and 5% CO_2_. After 5 min of stabilization period, the optimal length of the muscle was measured after two tetanic contractions (1 A, 300-ms duration, and 150-Hz stimulation frequency) with 5-min intervals. The Fo output was measured at the optimal length. The muscle was stimulated by one electronic stimulus with 1-min intervals. The Ft was detected by stimulation at 80 Hz for 300 ms with 1-min intervals. After measurements were conducted, the gastrocnemius was dried and weighed. The SFo and SFt were normalized with the muscle weight and optimal length (*55*). The data were analyzed using 611A Dynamic Muscle Analysis software.

### Quantitative real-time PCR

C2C12 myoblasts were collected using TRIzol for RNA extraction. The muscles were harvested and homogenized for RNA extraction using mortar and TRIzol (Invitrogen, 15596026). Then, 1 μg of RNA was subjected to reverse transcription to cDNA using the PrimeScript Real-Time Master Mix (TaKaRa, Japan). Power SYBR Green PCR Master Mix (Thermo Scientific) was applied for the qPCR of the target mRNA detection. The relative expression level of the gene of interest normalized to β-actin was calculated in accordance with the 2-ΔΔCT formula. The sequences of the primers used for real-time PCR assay are listed in table S1. The cycling conditions were denaturation at 95°C for 10 min, 40 cycles at 95°C for 15 s, optimal annealing temperature for 20 s, and 72°C for 30 s.

### Data analysis

The recorded microscopic images were processed and analyzed using Fiji-ImageJ (https://imagej.net/software/fiji/). The data were presented as means ± SD, with *n* as the number of cells or animals. The presentation of the system and in vitro experiments were all based on three biological replicates, and for each individual in vitro experiment, at least four technical replicates were analyzed. One-way or two-way analysis of variance (ANOVA) with appropriate post hoc tests was used for multiple-group comparisons. *P* < 0.05 was considered statistically significant. GraphPad Prism 7 was used for the above statistical analysis.
