# Supplementary material for: High-efficiency quantitative control of mitochondrial transfer based on droplet microfluidics and its application on muscle regeneration
Source: Sci Adv. 2022 Aug 17;8(33):eabp9245. doi: 10.1126/sciadv.abp9245 (PMC9385153; doi:10.1126/sciadv.abp9245)
Supplement: Supplementary file 1 — Supplementary Text Figs. S1 to S4 Table S1 [file sciadv.abp9245_sm.pdf]

Science Advances
AAAS

Supplementary Materials for

High-efficiency quantitative control of mitochondrial transfer based on droplet microfluidics and its application on muscle regeneration

Jiayu Sun *et al.*

Corresponding author: Dong Sun, medsun@cityu.edu.hk; Wayne Y. W. Lee, waynelee@cuhk.edu.hk

Sci. Adv. **8**, eabp9245 (2022)
DOI: 10.1126/sciadv.abp9245

The PDF file includes:

Supplementary Text
Figs. S1 to S4
Table S1
Legends for movies S1 to S3

Other Supplementary Material for this manuscript includes the following:

Movies S1 to S3

Supplementary Text

<u>Improvement of cell encapsulation efficiency of the developed system</u>

Single-cell encapsulation efficiency is a key indicator of the performance of a droplet microfluidic system aiming for single cell analysis. In theory, the cell encapsulation distribution follows the Poisson distributions as follows:

$$p(k,\lambda) = \frac{\lambda^k e^{-\lambda}}{k!}$$

where λ is the average number of cells per droplet and k is the cell number encapsulated in one droplet. Increasing the single cell encapsulation efficiency while reducing multiple-cell encapsulation efficiency is the basic requirement. Although an extra droplet sorting module could help to achieve this goal, the complexity and cost of the system would increase considerably. Compared with the droplet sorting module, the proposed wave-like structure before the droplet generation site helped to achieve this without increasing the complexity and cost of the developed system. It increased the single-cell encapsulation efficiency to 47.8% and decreased the multiple-cell encapsulation efficiency to 5.9% when the average number of cells per droplet was 0.6 (Fig. S1A). The single to multiple cells encapsulated ratio was improved to approximately 8.1, which was approximately 292% of the Poisson distribution when $\lambda = 0.6$, upon using the wave-like structure (Fig. S1B). Therefore, the developed wave-like structure-based system could perform considerably better than the traditional one-dimension droplet generation chip without the wave-like structure. Fig. S1C shows the cell focused state of the randomly incoming cells from the inlet within the wave-like structure, as recorded by a high-speed camera (MIKROTRON, i-SPEED 230). Compared with the flow rates of 100 $\mu L/30\ min$ and 200 $\mu L/30\ min$, the separation between cells was better at the flow rate of 300 $\mu L/30\ min$, and the multicellular encapsulation rate was reduced. Compared with the flow rate of 400 $\mu L/30\ min$, the distance between two adjacent cells was more suitable to reduce the ratio of none-cell encapsulated droplets and to improve the encapsulation ratio of single cells.

<u>Number of isolated mitochondria encapsulated in every droplet</u>

As discussed earlier, the isolated mitochondria (approximately 1 μm in diameter) were very small compared with the droplet (approximately 40 μm in diameter). Therefore, the isolated mitochondria were evenly encapsulated in each droplet and subjected to the isolated mitochondrial concentration. In this work, the number of isolated mitochondria encapsulated in one droplet was

counted in the 3D rebuilding image under a fluorescence confocal microscope (LEICA SP8LIA++ TRUE Confocal Laser Scanning Microscope). As shown in Fig. S2, using unit concentrations of 0.25, 0.5, and 1.0 of isolated-mitochondria suspension, 8, 22, and 41 isolated mitochondria were encapsulated in each droplet, respectively.

<u>Stability of transferred exogenous isolated mitochondria in recipient cell</u>

In the *in-vitro* experiments, the differentiation process of C2C12 myoblasts into C2C12 myotubes lasted for 7 days. In the *in-vivo* experiments, the healing process of $BaCl_2$i-induced muscle injury also lasted for 7 days. Therefore, the time that the transferred exogenous isolated mitochondria could exist in the recipient C2C12 cells was also investigated and referred to as stability here. Parts of the recipient C2C12 cells collected from the group with 1 unit concentration of exogenous isolated mitochondria were cultured for 7 days to investigate the stability of the transferred mitochondria. The exogenous isolated mitochondria stained with MitoTracker™ Green FM could be observed clearly on days 0 and 3, and on days 5 and 7 (Fig. S3), although the transferred mitochondria may have experienced division and apoptosis process.

<u>Wave-like structure focuses cells into a single line</u>

The loaded cells were randomly distributed at the inlet. As mentioned earlier, the wave-like structure can concentrate the randomly distributed cells into a line, and this concentrated line helps to improve the sing-cell encapsulation efficiency (Movie S1).

<u>Generation of droplets with high single-cell encapsulation efficiency</u>

By using the wave-like structure, the single-cell encapsulation efficiency was increased to 47.8% and the multi-cell encapsulation was reduced to 5.9%, with an average of 0.6 cell per droplet, beyond the Poisson distributions (Movie S2).

<u>Comparison of mitochondria movements in droplets and open environments</u>

Mitochondrial movement in droplets was restricted to a confined environment, such that mitochondria could have more opportunities to contact and be engulfed by recipient cells. In the open environment, mitochondria had more space to move. This reduced the chance of mitochondria reaching recipient cells (Movie S3).

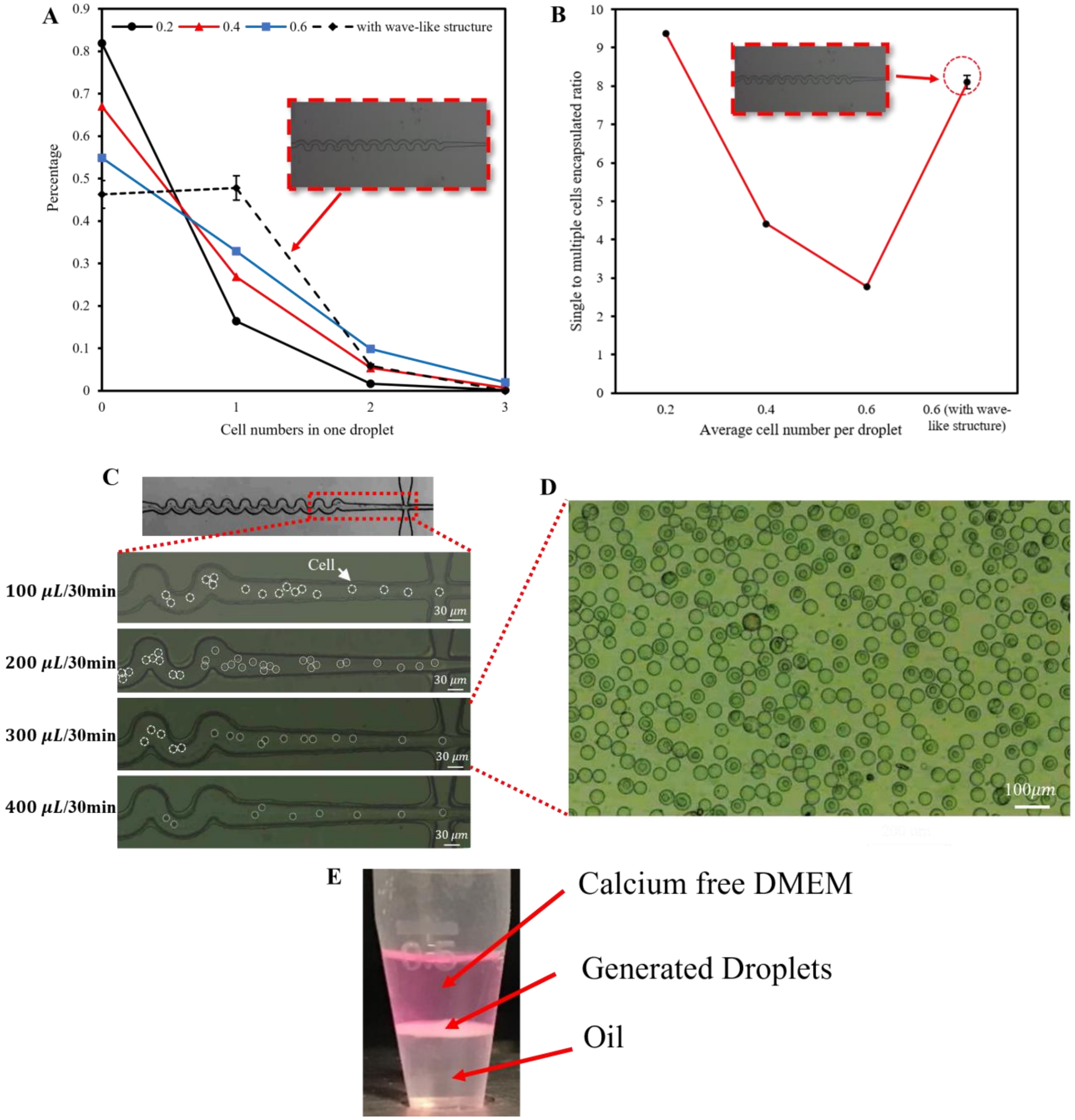

Fig. S1. Performance improvement of the developed system by using wave-like structure. (**A**) Poisson distribution of cell encapsulations in traditional droplet generation chip and the distribution of cell encapsulations in the developed system with the wave-like structure ($\lambda = 0.6$, flow rate of 300 μL/30 *min*). (**B**) The single-to-multiple cell encapsulation ratio was considerably improved using the wave-like structure. (**C**) Cell focused state in the wave-like structure under different cell suspension flow rates. (**D**) Generated droplets in the observation chip (with the wave-

like structure at a flow rate of 300 *μL/*30 *min*). (**E**) Generated droplets collected in the tube. The data of the wave-like structure are presented as mean ± SD.

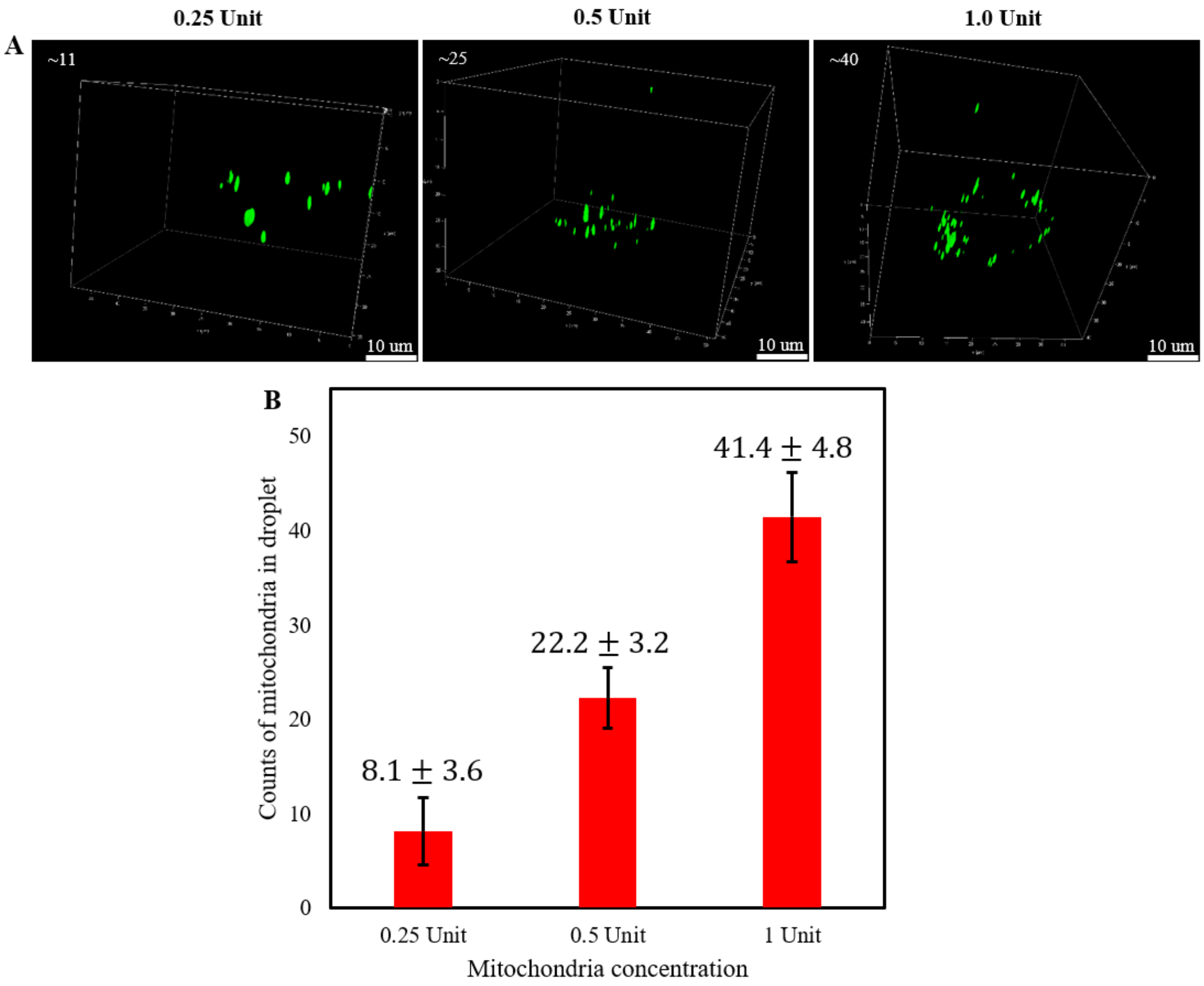

Fig. S2. Number of isolated mitochondria encapsulated in each droplet at different concentrations of isolated mitochondrial suspension. (**A**) 3D rebuilding images of isolated mitochondria (labelled with MitoTracker™ Green) encapsulated in one droplet at three different concentrations of isolated mitochondrial suspension (cells stained with Cell Mask Deep Red were hidden here by closing the red color channel of the confocal microscope). Every frame here contained one droplet, and the number of isolated mitochondria was labeled on the top left corner. (**B**) Number of isolated mitochondria encapsulated in each droplet at three different concentrations of isolated-mitochondria suspension. All data were presented as mean ± SD.

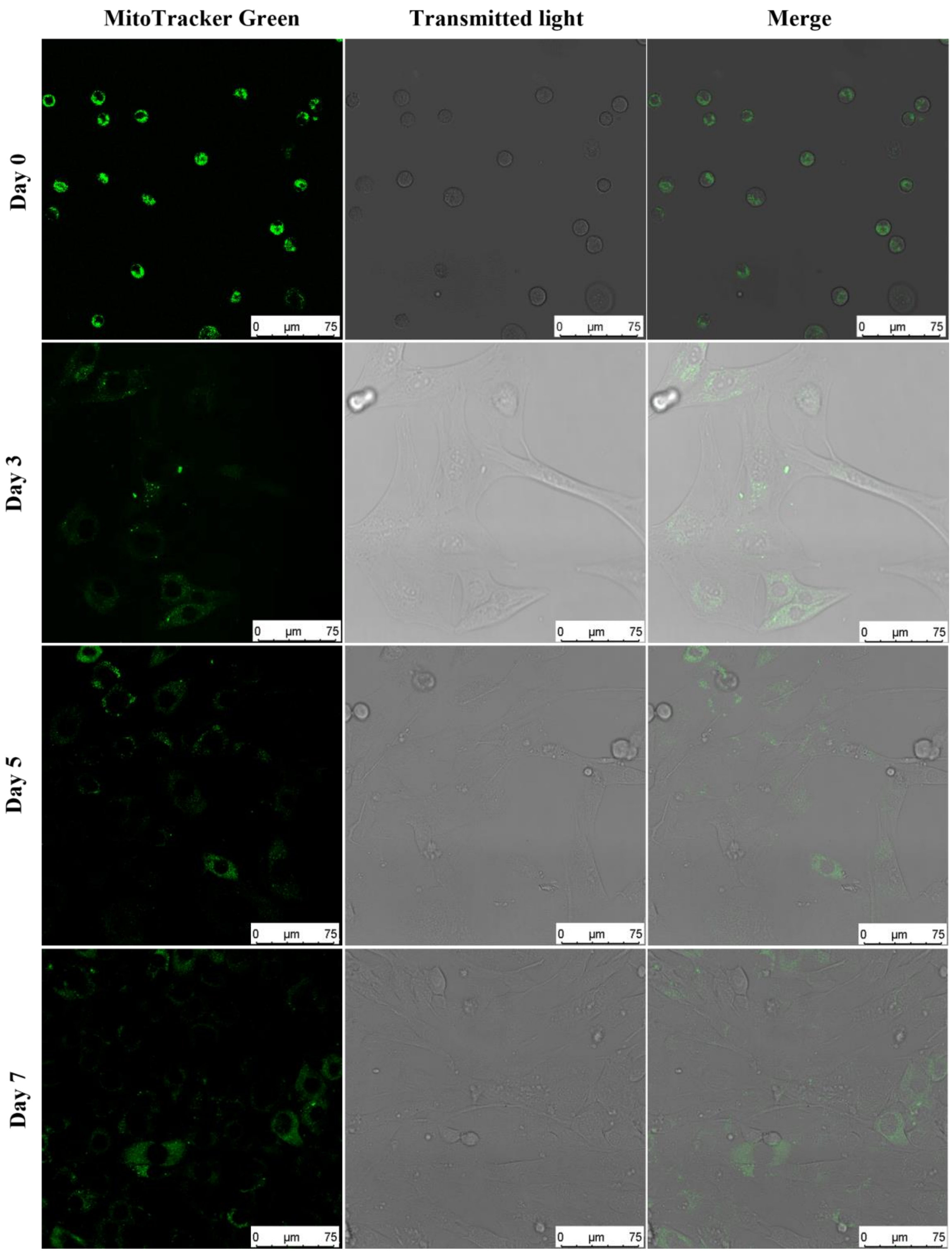

Fig. S3. Stability of transferred mitochondria in recipient cells (1 unit concentration of exogenous isolated-mitochondria suspension used). The green dots are the transferred mitochondria (stained with MitoTracker™ Green FM before isolation).

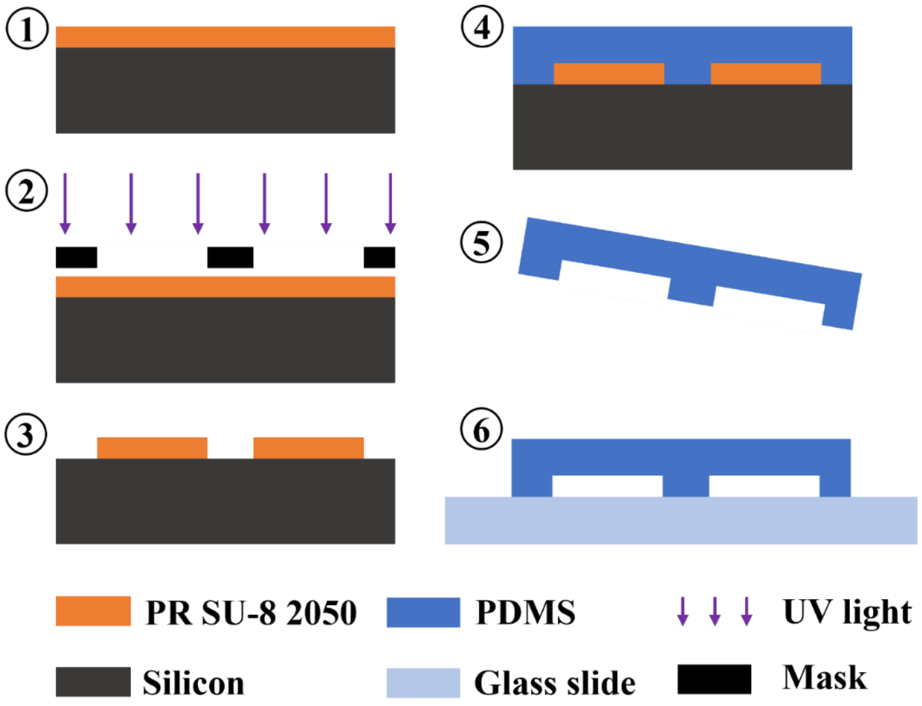

Fig. S4. Microfluidic chip fabrication using soft lithography. Step 1, spin coating of SU-8 2050 was performed on a silicon wafer at the height of 30 μm. **Step 2**, exposure was performed with a mask on the top of SU-8 2050 under ultraviolet light after pre-baking. **Step 3**, exposure SU-8 2050 was developed in the SU-8 developing reagent after post-baking. **Step 4**, PDMS (Polydimethylsiloxane) was solidified at 80 °C. **Step 5**, the cured PDMS was peeled off. **Step 6**, the cured PDMS was bonded to a glass slide.

Table S1. Sequence of primers for RT-qPCR

Sequence		Gene
Forward	TATGGAGTGACATAGAGTGTGCT	PGC-1a
Reverse	CCACTTCAATCCACCCAGAAAG	
Forward	GAGCCTTTTGCCAGCTTGTC	Sirt3
Reverse	AGCAGTTCTTGTGTCCACCC	
Forward	ATTCCGAAGTGTTTTTCCAGCA	mt-TFA
Reverse	TCTGAAAGTTTTGCATCTGGGT	
Forward	ATGTCGCTTTCCAACAAGCTG	Pgk1
Reverse	GCTCCATTGTCCAAGCAGAAT	
Forward	AGGAATGCCATCCGCTAC	Myf5
Reverse	CGTGATAGATAAGTCTGGAGC	
Forward	CTGCTCTGATGGCATGATGG	Myod1
Reverse	GTTCCCTGTTCTGTGTCGCT	
Forward	ATGGTGCCCAGTGAATGCAA	MyoG
Reverse	ACCCAGCCTGACAGACAATC	
Forward	CCCAAAGCTAACCGGGAGAAG	Acta1
Reverse	GACAGCACCGCCTGGATAG	
Forward	CAGACCGCAACCTAAACACA	CoxI
Reverse	TTCTGGGTGCCCAAAGAAT	
Forward	TAGAGGGACAAGTGGCGTTC	18s
Reverse	CGCTGAGCCAGTCAGTGT	
Forward	GGCTGTATTCCCCTCCATCG	β-actin
Reverse	CCAGTTGGTAACAATGCCATGT	

Movie S1. The wave-like structure focuses the randomly distributed cells from the inlet into a line at the outlet.

Movie S2. Generated droplets with a high single-cell encapsulation ratio of about 50%.

Movie S3. Comparison of isolated mitochondria moving in the droplet and open environment.
